# Orthostatic Hypotension Predicts Cognitive Impairment in the Elderly: Findings from a Cohort Study

**DOI:** 10.3389/fneur.2017.00121

**Published:** 2017-04-03

**Authors:** Haixia Huang, Tianheng Zheng, Fang Liu, Zhuoli Wu, Huazheng Liang, Shaoshi Wang

**Affiliations:** ^1^Department of Neurology, North Sichuan Branch of Shanghai No.1 People’s Hospital, Shanghai, China; ^2^Department of Neurology, The Affiliated Hospital of Guizhou Medical University, Guiyang, China; ^3^Neuroscience Research Australia, Randwick, NSW, Australia

**Keywords:** orthostatic hypotension, cognitive impairment, elderly, cohort study

## Abstract

**Background:**

Orthostatic hypotension (OH) is a known risk factor for cerebral ischemia, but its correlation with cognitive impairment (CI) is not well established.

**Objective:**

The aim of this study is to explore the relationship between OH and CI in the elderly.

**Methods:**

The study group consisted of 44 OH patients who presented with drowsiness, vertigo, and fatigue between January 2009 and December 2011 (OH group). Eighty-eight healthy elderly were paired with those in the OH group in a 1:2 based on their education levels (NOH group). Baseline sociodemographic information and cognition-related measures were collected for both groups. Cognitive function was assessed 4 years later using MMSE.

**Results:**

The overall incidence of CI was 14.0% among the 114 subjects who completed the follow-up assessment. There was a significant difference in the incidence of CI between the OH group (23.7%) and the NOH group (9.2%) (χ^2^ = 4.399, *P* = 0.036). After excluding the influence of age (OR = 1.199, 95% CI: 1.072–1.340, *P* = 0.001) and education years (OR = 0.568, 95% CI: 0.371–0.869, *P* = 0.009), OH (OR = 4.047, 95% CI: 1.144–14.313, *P* = 0.030) became an independent risk factor for CI.

**Conclusion:**

OH can lead to CI. We suggest that future studies, with a larger sample size, use OH exposure time instead of OH exposure population to verify the conclusion of this study.

## Introduction

Aging with the development of cognitive impairment (CI) has attracted extensive attention due to the increasing aging population (1–3). Studies have shown that hypertension is an independent risk factor for developing CI in the elderly (4, 5). However, other studies showed that hypotension can also lead to CI in the elderly (6, 7). Orthostatic hypotension (OH), a phenomenon of decreased blood pressure when changing from a supine position to the orthostatic position, is often accompanied by drowsiness, vertigo, blurred vision, and other signs of brain hypoperfusion. It is common in the elderly, with an incidence of 5–30% (8). Colloby et al. proposed that decreased systolic blood pressure (SBP) in the orthostatic position might be one of the causes of deep white matter hyperintensity (9). A recent study also reported that OH was an independent risk factor for ischemic stroke (10), which increased the incidence of senile dementia (8). Because this study focused on the effect of OH with baseline hypotension on cognitive performance, it is not conclusive that isolated OH leads to CI. This 7-year prospective cohort study aimed to investigate the relationship between OH and CI in the elderly.

## Subjects and Methods

### Subjects

Forty-four OH patients who visited our hospital between January 2009 and December 2011 due to drowsiness, vertigo, and fatigue were enrolled in the study group (OH group). Eighty-eight healthy elderly were paired with those in the OH group in a 1:2 based on their education levels (NOH group). The inclusion criteria were as follows: aged 60 years and older, secondary school or above education, fit the 1996 American Autonomic Society/American Academy of Neurology diagnostic criteria of OH [SBP dropped more than 20 mmHg or diastolic blood pressure (DBP) more than 10 mmHg when changed from the supine position to the orthostatic position], good understanding of questions or content of the questionnaire, no difficulty in communicating with researchers, and being conscious and able to give informed consent. Exclusion criteria were as follows: confirmed Alzheimer’s disease using the diagnostic criteria for probable AD dementia from NINCDS—ADRDA (11); vascular dementia; dementia due to other causes; confirmed multiple system atrophy; Parkinson’s disease; cerebral infarction; intracerebral hemorrhage; other cerebrovascular diseases; MMSE score less than 24; depression (using the Self-Rating Depression Scale); anxiety (using the Self-Rating Anxiety Scale); other psychiatric problems; traumatic injury to the head; visual and auditory deficits; hypertension; primary hypotension; postprandial hypotension; severe liver and kidney insufficiency; severe medical conditions such as pneumonia, coronary artery disease, and heart failure; cigarette and alcohol addiction; and abuse of medications. We have obtained ethical approval from the Human Research Ethics Committee of North Sichuan Branch, Shanghai No.1 People’s Hospital.

### Methods

#### Baseline Information

Baseline information was collected after obtaining the informed consent from the patients. It included sociodemographic information such as age, gender, education level, living alone or not, cognition-associated risk factors such as hypertension (5), smoking, presence of diabetes mellitus, supine SBP and DBP, head MRI, and Minimal Mental State Examination (MMSE). OH diagnosis was achieved by measuring supine blood pressure 2 min after lying in bed, followed by measuring the orthostatic blood pressure within 1–3 min after standing up quickly from the bed. BP was measured on the right arm, 3 cm above the cubital fossa, for 3 consecutive days at the same time each day. (The averages of results from the 3 consecutive days were compared.) The sphygmomanometer was kept *in situ* and on the same level as the patient’s heart. MR imaging was performed using a GE 1.5 T superconducting MRI. Horizontal T1WI, T2WI, and FLAIR were collected regularly, and in some cases, coronal and sagittal T2WIs were collected. White matter lesions (WMLs) were assessed by specialists using the standard suggested by Fazekas et al. (12): 0 indicates no or a single punctate lesion, 1 indicates multiple punctate lesions, 2 indicates fusion or bridging of multiple lesions, and 3 indicates extensive fusion of WML.

#### Follow-up Information

Four years later, these patients were assessed with Minimal Mental State Examination (MMSE), and CI was diagnosed by neurologists using the MMSE scale during the interview. MMSE is widely used for cognitive assessment, which examines attention, orientation, memory, calculation, language, and visual spacing capabilities of cognitively impaired patients. It has a total score of 30. If the score is between 24 and 27, it is very likely that the patients have mild cognitive impairment (MCI), and if it is below 24, then the patient might have dementia. If the patient had a complaint of cognitive decline or the caregiver reported that the patient had cognitive decline in less than 4 years, the patient would be interviewed by an experienced neurologist, and MMSE was used to assess the patient’s cognitive performance. If the score was lower than 24, the patient was diagnosed with CI (13).

#### Data Analysis

Original data were checked and recorded in Epidata 3.0, followed by checking errors. SPSS 13.0 was used to analyze the data. Categorical data were represented as frequency or percentage and analyzed using Chi-square test. Non-parametric testing was used to analyze the difference in baseline information. Quantitative data were represented as average ± SD, and the difference in baseline information between groups was analyzed using *t*-test. Logistic regression model was established to predict the probability of developing CI, and it was tested bilaterally. The significant difference was indicated when *P* was less than 0.05.

## Results

### Baseline Data

Of 44 OH patients and 88 education-level paired normal patients, 68 were men and 64 were women. Their age ranged from 60 to 87 years with an average of 69.58 ± 6.49 years. The average education time was 11.70 ± 2.04 years. The average SBP and DBP on a supine position were 137.08 ± 17.80 and 85.03 ± 11.48 mmHg, respectively. The percentage of patients living alone and smoking was 10.6 and 15.2%, respectively. Twenty-four had diabetes (18.2%). The percentage of WML 0, 1, 2, and 3 was 45.5% (60), 34.1% (45), 15.2% (20), and 5.3% (7), respectively. There was no significant difference in baseline demographic information and cognition-related risk factors between the OH group and the normal controls (*P* > 0.05) (Table 1).

**Table 1:** **Comparison of baseline demographic information and cognition-related risk factors between the orthostatic hypotension (OH) and non-OH groups ($\bar{x}\pm \text{s}$ or n (%))**.

Variables	Total subjects (132)	OH group (44)	Non-OH group (88)	*t*/χ^2^/*Z*	*P* value
Age	69.58 ± 6.49	69.90 ± 6.03	68.92 ± 7.34	0.819	0.414
Male	68 (51.5)	18 (40.9)	50 (56.8)	1.370	0.124
Education (years)	11.70 ± 2.04	11.78 ± 2.09	11.52 ± 1.95	0.693	0.489
Living alone	14 (10.6)	4 (9.1)	10 (11.36)	0.160	0.690
Smoking	20 (15.2)	6 (13.6)	14 (15.9)	0.118	0.731
Diabetes	24 (18.2)	10 (22.7)	14 (15.9)	0.917	0.338
Supine systolic blood pressure (SBP)	137.08 ± 17.80	138.92 ± 18.04	135.86 ± 17.78	0.928	0.355
Orthostatic SBP	115.45 ± 17.41	92.23 ± 20.02	125.38 ± 17.01	9.941	<0.001
Supine diastolic blood pressure (DBP)	85.03 ± 11.48	86.01 ± 11.63	83.67 ± 11.19	1.118	0.266
Orthostatic DBP	72.82 ± 11.75	65.70 ± 14.12	80.16 ± 10.08	6.767	<0.001
MMSE	26.66 ± 1.16	26.41 ± 1.26	26.78 ± 1.09	1.768	0.079
White matter lesion category				1.580	0.115
0	60 (45.5)	15 (34.1)	45 (51.1)		
1	45 (34.1)	19 (43.2)	26 (29.5)		
2	20 (15.2)	7 (15.1)	13 (14.8)		
3	7 (5.3)	3 (6.8)	4 (4.5)		

### Comparison of Baseline Information between Follow-up and Non-Follow-up Groups

Among the 44 paired groups, 6 groups were not followed up for various reasons: 1 group had a death, 3 groups refused being followed up, and 2 groups lost contact. It was found that the education level of these 6 groups was lower than the other 38 groups (*t* = 4.016, *P* < 0.001). There was no significant difference in other baseline information between them (*P* > 0.05) (Table 2).

**Table 2:** **Comparison of baseline demographic information and cognition-related risk factors between followed up group and non-followed up group ($\bar{x}\pm \text{s}$ or n (%))**.

Variables	Non-followed up group (6 groups)	Followed up group (38 groups)	*t*/χ^2^/*Z*	*P* value
Age	71.53 ± 5.68	69.27 ± 6.57	1.382	0.169
Male	8 (44.4)	60 (52.6)	0.417	0.518
Education (years)	10.00 ± 1.37	11.96 ± 2.00	4.016	<0.001
Living alone	2 (11.1)	12 (10.5)	0.114	0.736
Smoking	4 (22.2)	16 (14.0)	0.298	0.585
Diabetes	5 (27.8)	19 (16.7)	0.651	0.420
Supine systolic blood pressure (SBP)	139.23 ± 19.45	137.12 ± 17.78	0.462	0.645
Orthostatic SBP	116.15 ± 16.25	114.32 ± 16.02	0.450	0.654
Supine diastolic blood pressure (DBP)	84.13 ± 10.32	86.25 ± 11.93	0.713	0.478
Orthostatic DBP	72.00 ± 10.82	72.88 ± 11.97	0.293	0.770
MMSE	26.78 ± 1.40	26.64 ± 1.12	0.467	0.642
White matter lesion category			0.521	0.615
0	7 (38.9)	53 (46.5)		
1	7 (38.9)	38 (33.3)		
2	3 (16.7)	17 (14.9)		
3	1 (5.5)	6 (5.3)		

### Comparison of MMSE between OH and Non-OH Groups

Among the 114 subjects who completed the follow-up assessment, there was no significant difference in baseline MMSE score between the OH and non-OH groups. After 4 years, there was a significant difference between these two groups (*t* = 2.441, *P* = 0.018). Within each group, the MMSE score also showed a significant decline (both *P* < 0.005) (Table 3).

**Table 3:** **Comparison of MMSE score between orthostatic hypotension (OH) and non-OH groups**.

Variables	OH group (38)	Non-OH group (76)	*t*	*P* value
Baseline	26.30 ± 1.33	26.48 ± 1.22	0.720	0.472
Follow-up	24.08 ± 3.47	25.58 ± 2.16	2.441	0.018
*t*	3.683	3.163		
*P* value	<0.001	0.002		

### Single-Factor Analysis for CI

Sixteen subjects were diagnosed with CI (incidence of 14.0%) with 9 in the OH group (38 in total, incidence 23.7%) and 7 in the non-OH group (total 76, incidence 9.2%). There was a significant difference in the incidence of CI (χ^2^ = 4.399, *P* = 0.036). These subjects were further divided into two groups based on whether they developed CI. Variables such as age, gender, education years, living alone or not, smoking, diabetes, WML category, and OH were compared between groups. We found that there was a significant difference between the two groups in age (*t* = 2.958, *P* = 0.004) and OH (χ^2^ = 4.399, *P* = 0.036) (Table 4).

**Table 4:** **Single-factor analysis for cognitive impairment (CI) between CI and non-CI groups**.

Variables	CI group (16)	Non-CI group (98)	*t*/χ^2^/*Z*	*P* value
Age (years)	73.63 ± 8.43	68.56 ± 5.97	2.958	0.004
Male	10 (62.5)	50 (51.0)	0.727	0.394
Education (years)	10.00 ± 1.37	11.96 ± 2.00	4.016	<0.001
Living alone	2 (12.5)	10 (10.2)	0.077	0.781
Smoking	2 (12.5)	14 (14.3)	0.036	0.849
Diabetes	4 (25.0)	15 (15.3)	0.931	0.335
White matter lesion category				1.580
0	7 (43.8)	46 (46.9)	2.454	0.484
1	4 (25.0)	34 (34.7)		
2	3 (18.8)	14 (14.3)		
3	2 (5.3)	4 (4.1)		
OH	9 (56.3)	29 (29.6)	4.399	0.036

### Multifactor Analysis for CI

By taking CI as the dependent variable and age, education, and OH as independent variables, logistic regression analysis for multiple variables showed that OH (OR = 4.047, 95% CI: 1.144–14.313, *P* = 0.030) was an independent risk factor for CI after excluding the influence of age (OR = 1.199, 95% CI: 1.072–1.340, *P* = 0.001) and education years (OR = 0.568, 95% CI: 0.371–0.869, *P* = 0.009). Based on the predicted probability from the logistic regression model, an ROC curve was mapped. The area under the ROC curve was 0.816 (95% CI: 0.710–0.922). The maximum point of Youden’s index was considered as the cutoff point, and the prediction probability was 0.172 at this point. The sensitivity was 81.30%, the specificity was 77.60%, and the Youden’s index was 0.589 (Table 5).

**Table 5:** **Multifactor analysis for cognitive impairment**.

Variables	*B*	SE	Wald χ^2^	*P*	OR	95% CI for OR	
						Lower	Upper
Orthostatic hypotension	1.398	0.645	4.704	0.030	4.047	1.144	14.313
Age	0.181	0.057	10.177	0.001	1.199	1.072	1.340
Education (years)	−0.566	0.217	6.798	0.009	0.568	0.371	0.869

## Discussion

Studies have shown that hypotension is an independent risk factor for dementia, with brain hypoperfusion proposed to account for this (14, 15). But the relationship between OH and CI has not been studied in longitudinal studies. This study aimed to study this relationship by comparing 44 OH patients with 88 healthy subjects after 4 years follow-up. Among the patients who were followed up, 23.7% of OH patients developed CI, whereas only 9.2% of non-OH subjects developed this illness. The difference was significant. Furthermore, OH still remains an independent risk factor for CI even after excluding the influence of age and education years, indicating that increased blood pressure variance is related to the development of CI in the elderly. In this study, the subjects who were not followed up had lower education level, which was demonstrated to be a risk factor for CI in another study (6). Our findings are consistent with their results.

Results of this study are consistent with that of previous cross-sectional studies (16–19). However, cross-sectional studies can only provide a clue to the etiology of a disease, whereas longitudinal studies can reveal causation.

Elmstáhl and Rosén (20) reported their results about OH and CI in a longitudinal study. They monitored the EEG change among healthy females, which reflected the causal relationship between OH and CI (20). To our knowledge, this is the first study to investigate the relationship between OH and CI. Not all subjects in the study by Elmstáhl and Rosén fit the diagnostic criteria of OH, which may have impacted their findings. This study found that age is an independent risk factor for CI. As age increases, the likelihood of developing CI also increases, which is consistent with the previous findings. Multiple mechanisms might explain the relationship between CI and OH. Brain areas that are responsible for CI might regulate cardiovascular activities. Hence, neurodegeneration of these brain areas may be the cause of CI and OH (21). Studies have reported that OH leads to hypoperfusion of the frontal lobe, which could result in compromised executive functions of patients (22–25). Finally, similar to the relationship between hypotension and CI, long-term brain hypoperfusion caused by the large variance of blood pressure in OH patients leads to ischemic demyelinating WMLs, which accounts for the impaired cognition (26, 27).

This study used MMSE to assess the cognitive performance of all subjects. The reason for this is that MMSE is widely used in the field of dementia research and other batteries which are popular in the western world have not been validated for Chinese people due to culture difference. MMSE has a high sensitivity and specificity and has been widely used in clinical trials, and we believe that it can be a good tool for cognitive assessment.

There are some limitations in this study. First, the sample size is small due to our strict exclusion criteria, which might lead to the underestimated relationship between WML and CI. Second, development of OH in the control group was not considered during the follow-up period. So we suggest that future studies should increase the sample size and also use OH exposure time instead of OH exposure population to confirm the conclusion of this study. Third, this study used the MMSE score of 24 as the cutoff point for CI. A small number of our patients had a score between 24 and 27, which indicates that they had MCI when they were recruited. It is known that MCI has an annual conversion rate of 10.24% for dementia (28). Therefore, future large-scale studies can stratify their research design to include both MCI with OH and pure OH patients to clarify the relationship between OH and CI.

## Ethics Statement

This study was carried out in accordance with the recommendations of “Chinese guidelines in human research, The Human Research Ethics Committee of North Sichuan Branch, Shanghai No.1 People’s Hospital, Shanghai Jiaotong University,” with written informed consent from all subjects. All subjects gave written informed consent in accordance with the Declaration of Helsinki. The protocol was approved by the “The Human Research Ethics Committee of North Sichuan Branch, Shanghai No.1 People’s Hospital, Shanghai Jiaotong University.”

## Author Contributions

SW, HL, and HH initiated this study. HH, TZ, ZW, and FL collected the clinical data and did statistical analysis. HL wrote this manuscript.

## Conflict of Interest Statement

The authors declare that the research was conducted in the absence of any commercial or financial relationships that could be construed as a potential conflict of interest.
